# BiSpectral Index (BIS) monitoring may detect critical hypotension before automated non-invasive blood pressure (NIBP) measurement during general anaesthesia; a case report.

**DOI:** 10.12688/f1000research.3-5.v1

**Published:** 2014-01-09

**Authors:** Matthew M. J. Smith

**Affiliations:** 1 Department of Anaesthetics, Sheffield Teaching Hospitals, Sheffield, S10 2JF, UK

## Abstract

A patient undergoing general anaesthesia for neurosurgery exhibited an unexpected sudden decrease in the BiSpectral Index (BIS) value to near-zero. This prompted the detection of profound hypotension using non-invasive blood pressure (NIBP) measurement and expedited urgent assessment and treatment, with the patient making a full recovery. Widely regarded as a ‘depth of anaesthesia’ monitor, this case demonstrates the potential extra clinical benefit BIS may have in the detection of critical incidents such as anaphylaxis during general anaesthesia.

## Presentation and clinical findings

A retired, 68 year old man of white British origin presented to the neurosciences unit with a subdural haematoma of unknown aetiology. With no past medical or family history of note, he was scheduled for neurosurgical intervention via burr holes under general anaesthesia. Prior to the operation, his Glasgow Coma Score was 15/15 and he was alert and oriented. The patient was in good general health, normotensive, and had no regular medications or known drug allergies. A total intravenous anaesthesia (TIVA) technique was chosen, as is usual for such cases in our institution.

Before induction of anaesthesia, we instituted standard monitoring according to guidelines published by the Association of Anaesthetists of Great Britain and Ireland. A BIS Quattro sensor (Covidien LLC, Mansfield, USA) was also applied to the forehead on the non-pathological side and connected to a BIS ‘VISTA’ monitor (Covidien LLC, Mansfield, USA).

Target-controlled infusions of propofol (3µg/ml) and remifentanil (3ng/ml) were used to induce unconsciousness. To maintain normotension, a 4mg/h infusion of metaraminol was simultaneously started. Once the BIS value had fallen to 60, a 40mg dose of atracurium was given to facilitate tracheal intubation. During this period cardiovascular stability was maintained as measured by pulse rate and regular (every 2.5m) NIBP readings.

The patient was prepared for the operating theatre and transferred into the operating room, where we noticed that the BIS value had dramatically fallen to 04 with an almost isoelectric real-time EEG reading. This triggered the anaesthetist to immediately re-measure the NIBP, which revealed a blood pressure of 44/26.

Possible anaphylaxis to atracurium was suspected. Immediate treatment was initiated with 250ml 0.9% saline, 6mg ephedrine and 0.5mg of metaraminol IV, whilst adrenaline was prepared. These interim measures were enough to restore the blood pressure and BIS back to their expected values, and in the event no adrenaline was administered. Subsequent acute care included institution of invasive blood pressure monitoring, and treatment with hydrocortisone 100mg, chlorphenamine 10mg, and ranitidine 50mg IV. Moderate flushing and urticaria became evident some 15 minutes after the onset of the hypotension, however the patient remained otherwise stable and the rest of surgery and recovery were uneventful. Blood samples for mast-cell tryptase were taken as per local guidelines.

## Timeline

**Figure 1.**
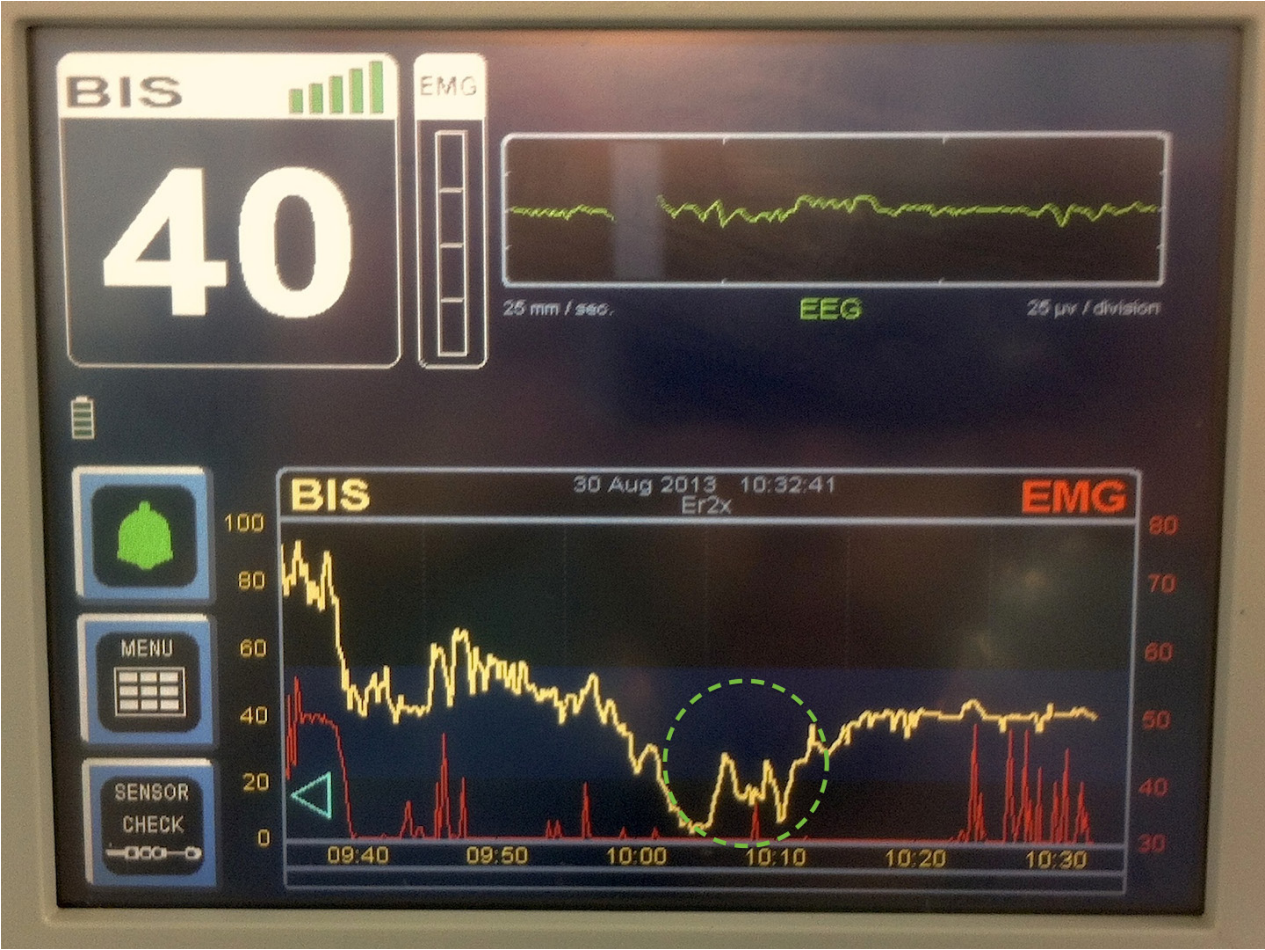
BIS Vista display illustrating the timeline and BIS trend during the period of hypotension.

Figure 1 below illustrates the timeline of the case. Anaesthetic induction occurs at 0940. The period of low BIS and hypotension is encircled in dashed green. The subsequent rise in BIS was coincident with restoration of normotension.

## Diagnosis and follow up

The patient had an uncomplicated post-operative recovery and was discharged and sent home. He was referred to the immunology clinic for follow-up allergy testing. Mast cell tryptase results were all within the norm, making suspicion of anaphylaxis unlikely; however a raised IgE (147IU/L) plus the clinical signs seen during anaesthesia raised the possibility of an anaphylactoid drug reaction. A non-immune-mediated cause could not be ruled out for the critical incident.

## Discussion

BIS uses the frontal electroencephalogram and proprietary algorithms to quantify level of consciousness on a scale of arbitrary units from 0 (isoelectric raw signal) to 100 (awake). Intended to guide the hypnotic aspect of general anaesthesia, previous reports have however suggested that BIS may be used as a crude marker of cerebral hypoperfusion ^1– 5^. Positron Emission Tomography scanning of anaesthetised patients has correlated the cerebral metabolic rate for oxygen consumption with BIS ^6^, strengthening the case for this.

During the events described, the drop in BIS was detected before that of any other monitored variable or clinical change. It prompted rapid reassessment, discovery of cardiovascular collapse, and timely intervention. Had the patient not been BIS monitored, the hypotension would not have been revealed until the next ‘scheduled’ cycling of the NIBP, which may have led to further harm. We feel this demonstrates the potential extra clinical utility that BIS may have in the detection of critical incidents over and above its perception as solely a ‘depth of anaesthesia’ monitor.

## Consent

Informed written consent for publication of clinical details and clinical images was obtained from the patient at the time of allergy clinic referral.
