# Supplementary material for: BiSpectral Index (BIS) monitoring may detect critical hypotension before automated non-invasive blood pressure (NIBP) measurement during general anaesthesia; a case report
Source: F1000Res. 2014 Jan 9;3:5. [Version 1] doi: 10.12688/f1000research.3-5.v1 (PMC4038321; doi:10.12688/f1000research.3-5.v1)
Supplement: Peer review history

# Peer review: Referee response for version 1

**Published:** 2014-03-25

**Authors:** Sumio Hoka

**Affiliations:** 1 Department of Anesthesiology and Critical Care Medicine, Kyushu University, Fukuoka, Japan

This is an interesting case report showing an immediate response of BIS monitor to circulatory failure.  BIS monitors could express not only the depth of anesthesia but also the state of brain ischemia.

I have read this submission. I believe that I have an appropriate level of expertise to confirm that it is of an acceptable scientific standard.

# Peer review: Referee response for version 1

**Published:** 2014-01-17

**Authors:** Ehab Farag

**Affiliations:** 1 Department of General Anesthesiology, Cleveland Clinic, Cleveland, OH, USA

It is a nice case, I approve this with no reservations.

I have read this submission. I believe that I have an appropriate level of expertise to confirm that it is of an acceptable scientific standard.
